# Transitioning adolescents with rare forms of diabetes to adult care: challenges and perspectives

**DOI:** 10.1530/EC-25-0451

**Published:** 2025-11-11

**Authors:** Felix Reschke, Gundula Ernst, Olga Kordonouri, Sarah K Lyons, Barbara Piccini, Andrea M Isidori, Ulla Döhnert, Rebecca Toenne, Francesca Dassie, Evangelia Charmandari, Violeta Iotova, Jantje Weiskorn, Valeria Corradin, Nolwen LeFloch, Bernd Rosenbichler, Pietro Maffei, on behalf of the Endo-ERN Transition of Care Working Group & TALENT Initiative

**Affiliations:** ^1^ Center for Endocrinology, Diabetology & Clinical Research, Children’s Hospital AUF DER BULT, Hannover, Germany; ^2^ Betreuungsnetz, Network for the Care of Seriously Ill Children, Adolescents and Young Adults, Hannover, Germany; ^3^ Hannover Medical School, Department of Medical Psychology, Hannover, Germany; ^4^ Division of Pediatric Diabetes and Endocrinology, Baylor College of Medicine and Texas Children’s Hospital, Houston, USA; ^5^ Endocrinology and Diabetology Unit, Meyer University Children’s Hospital IRCCS, Florence, Italy; ^6^ Department of Experimental Medicine, Sapienza University of Rome, Endo-ERN Centre “Azienda Ospedaliero-Universitaria Policlinico Umberto I”, Rome, Italy; ^7^ Division of Paediatric Endocrinology and Diabetes, Department of Paediatrics and Adolescent Medicine, University of Lübeck, Lübeck, Germany; ^8^ Department of Medicine (DIMED), Padua University, Padua, Italy; ^9^ Division of Endocrinology, Metabolism and Diabetes, First Department of Pediatrics, National and Kapodistrian University of Athens Medical School, ‘Aghia Sophia’ Children’s Hospital, Athens, Greece; ^10^ Department of Pediatrics, Medical University Varna, Varna, Bulgaria; ^11^ Italian Association for Lipodystrophies, Dossobuono, Italy; ^12^ Association du Syndrome de Wolfram (ASW), Grand-Champ, France; ^13^ Alstrom Syndrom e.V, Unterföhring, Germany

**Keywords:** rare diabetes, transition of care, adolescents and young adults, multidisciplinary care, monogenic diabetes, patient-centered care

## Abstract

**Background:**

Adolescents and young adults (AYA) with rare forms of diabetes – including Wolfram syndrome (WS), Alström syndrome (AS), Bardet-Biedl syndrome (BBS), and maturity-onset diabetes of the young (MODY) – face unique challenges during the transition to adult care. These challenges are intensified by multisystem endocrine involvement, neurocognitive and sensory impairments, and limited adult provider expertise.

**Objective:**

This narrative review describes transition-specific barriers in rare diabetes syndromes, explores current initiatives, and proposes recommendations for care models and health system reform.

**Key issues:**

Syndromic forms of diabetes often involve complex endocrine dysfunctions beyond glycemic control, including diabetes insipidus, hypogonadism, and thyroid or pituitary anomalies. Transitions are further hindered by diagnostic uncertainty, fragmented care structures, and insufficient interdisciplinary coordination. Pediatric care is often proactive and family-centered, while adult services are fragmented and reactive. Dedicated multidisciplinary transition services remain scarce.

**Recommendations:**

Best practices include early transition planning, syndrome-specific education, the use of patient-reported outcome measures (PROMs), and integration of digital tools. Structured collaboration between pediatric and adult providers – including virtual models – should be supported. Patient-centered approaches must address both medical and psychosocial readiness, with tailored communication for those with sensory or cognitive impairments.

**Health system and policy needs:**

Sustainable transition programs require dedicated funding, institutional prioritization, and policy inclusion in national and European rare disease frameworks. Without adequate financial support, disparities in care continuity and outcomes are likely to persist.

**Conclusion:**

A coordinated, multidisciplinary, and resourced transition model is essential to safeguard health, autonomy, and long-term outcomes in AYA with rare diabetes syndromes.

**Plain language summary:**

Young people with rare forms of diabetes – such as Wolfram syndrome (WS), Alström syndrome (AS), Bardet-Biedl syndrome (BBS), or maturity-onset diabetes of the young (MODY) – face special challenges when moving from pediatric to adult healthcare. These rare conditions often affect more than just blood sugar and can involve vision, hearing, and other parts of the body. As they grow older, these adolescents must not only manage their complex health needs but also learn to take more responsibility for their care. This article explains why the transition to adult care is especially difficult for this group. It shares experiences from families and healthcare providers and describes what can help: early preparation, teamwork between child and adult doctors, digital tools, and emotional support. The authors call for stronger guidelines and better cooperation across healthcare systems so that young people with rare diabetes can stay healthy and feel supported during this important time in life.

**Graphical abstract:**

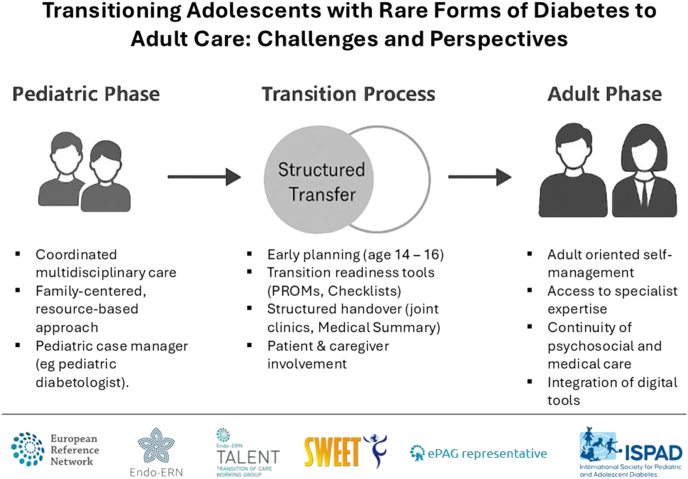

## Introduction

The transition from pediatric to adult care represents a critical phase in the continuum of healthcare, particularly for persons with chronic and rare diseases. Transition is defined as a purposeful, planned process that addresses the medical, psychosocial, and educational needs of adolescents as they move from child- to adult-centered healthcare systems (1). While often used interchangeably, transition and transfer represent conceptually distinct processes in healthcare. Transfer refers to the specific event in which care responsibility shifts from pediatric to adult providers, typically occurring during a single consultation or short time window. In contrast, transition is a structured, longitudinal process that begins well before the actual transfer and extends into post-transfer care, focusing on fostering autonomy, continuity, and psychosocial preparedness in adolescents with chronic diseases (2, 3).

Several national and international guidelines provide structured, cross-condition recommendations for the transition from pediatric to adult healthcare. The NICE guideline NG43 on transition (4) emphasizes early planning, developmentally appropriate education, involvement of parents and caregivers, and the importance of mental health screening. Similarly, White *et al.* (5) and Pape & Ernst (6) advocate for structured, individualized transition pathways supported by digital tools and coordinated multidisciplinary care.

While these approaches have been increasingly adopted for more common chronic conditions, such as type 1 diabetes, young people with rare forms of diabetes – including maturity-onset diabetes of the young (MODY), Alström syndrome (AS), Bardet-Biedl syndrome (BBS), and Wolfram syndrome (WS) – remain significantly underserved. These conditions are frequently associated with multisystem involvement, neurocognitive impairments, and progressive complications, which complicate standard transition models (7, 8).

In practice, most transition pathways fail to address the complexity of syndromic diabetes. For example, sensory or intellectual disabilities may limit adolescents’ ability to independently manage care, and adult specialists are often unfamiliar with rare syndromes. Therefore, translating best practices – such as those outlined in national guidance – into disease-specific transition models is crucial. This includes tailored patient education, formal caregiver inclusion, validated transition readiness tools, and digital platforms that enable care continuity and cross-specialty communication (9, 10).

Rare diabetes syndromes often involve complex multisystemic manifestations (e.g., retinal dystrophy, deafness, renal and cardiac anomalies, and cognitive impairments), requiring lifelong multidisciplinary care. This complexity, combined with diagnostic uncertainty, limited disease-specific expertise among adult care providers, and the absence of harmonized care protocols, poses significant challenges to successful transition (11, 12). As Stepien *et al.* (8) demonstrated in a European survey on rare metabolic disorders, transition services are often fragmented, characterized by inconsistent policies, poor documentation, and lack of communication between pediatric and adult services.

The recent literature further highlights the heightened risk of adverse outcomes during transition for individuals with rare conditions, including deterioration in metabolic control, loss to follow-up, and psychosocial distress (13, 14). Specific barriers include insufficient knowledge among adult endocrinologists, absence of specialized transition clinics, and lack of validated patient-reported outcome measures (PROMs) tailored to this group.

Despite these advances, actionable models for rare diabetes transition remain scarce. This review aims to synthesize the current state of transition care in MODY, AS, WS, and BBS, identify existing challenges, and propose evidence-informed strategies to optimize outcomes and continuity of care.

While this review focuses on selected syndromic forms of rare diabetes, such as WS, AS, BBS, and MODY, it is important to acknowledge that diabetes also occurs in the context of other rare conditions, including syndromes characterized by severe insulin resistance (e.g., congenital generalized lipodystrophy and INSR mutations) or autoimmune polyendocrine syndromes (e.g., IPEX and APS-1).

## Clinical characteristics and transition challenges in rare diabetes syndromes

Adolescents and young adults (AYA) with rare diabetes syndromes face a complex interplay of medical, psychosocial, and structural challenges when transitioning from pediatric to adult care. These conditions – such as MODY, WS, AS, and BBS – are characterized by early-onset diabetes, often combined with multisystem complications that require long-term interdisciplinary coordination. The transition period is particularly vulnerable due to gaps in disease-specific knowledge among adult providers, limited access to multidisciplinary services, and psychosocial barriers related to autonomy and self-management.

### Syndrome-specific features and transition implications

The clinical profiles and transition-specific challenges in rare diabetes syndromes are summarized in Table 1.

- MODY refers to a group of monogenic diabetes subtypes caused by autosomal dominant mutations affecting pancreatic beta-cell function. Children with MODY are often misdiagnosed with type 1 or type 2 diabetes, which leads to inappropriate insulin use and lack of genetic confirmation. Without a precise diagnosis, adult services may fail to adjust treatment (e.g., switching from insulin to sulfonylureas) or recognize familial implications (15, 16).
- WS is a progressive neurodegenerative disorder combining insulin-dependent diabetes mellitus, optic atrophy, sensorineural hearing loss, diabetes insipidus, and neurologic decline. The transition must accommodate increasing sensory and cognitive impairments and involve specialties such as neurology, ophthalmology, audiology, and nephrology. Mental health and family planning services are also essential (17, 18).
- AS features severe insulin resistance, obesity, cone-rod dystrophy, hearing loss, cardiomyopathy, and renal impairment. Transition is hindered by progressive visual and auditory deficits, necessitating the use of adaptive technologies and ongoing caregiver involvement (19, 20).
- BBS is characterized by obesity, retinal dystrophy, cognitive impairment, renal anomalies, and polydactyly. Intellectual disability and behavioral disorders can affect readiness for autonomous care. Transition plans must therefore include tailored education, literacy-adjusted communication, and the engagement of social workers or case managers (21).

**Table 1:** Clinical profiles and transition-specific challenges in rare diabetes syndromes. This table outlines four rare diabetes syndromes – MODY, WS, AS, and BBS – focusing on their key clinical characteristics, transition-related barriers, and recommended considerations for care continuity. The distinct syndromic features emphasize the need for individualized, multisystem transition planning and tailored patient education strategies.

Syndrome	Key clinical features	Transition challenges	Special considerations
MODY	Monogenic diabetes, often autosomal dominant; varying treatment needs depending on subtype	Often misdiagnosed; lack of genetic confirmation; misclassification affects treatment	Ensure molecular diagnosis; educate adult providers on subtype-specific management; discuss family implications
Wolfram syndrome	Diabetes mellitus, optic atrophy, diabetes insipidus, deafness, neurodegeneration	Dual sensory impairment; cognitive decline; multisystem care needs	Coordinate multidisciplinary transition planning; monitor psychological wellbeing; offer genetic counseling
Alström syndrome	Insulin resistance, cone-rod dystrophy, sensorineural hearing loss, cardiomyopathy	Progressive sensory impairment; caregiver dependency	Provide accessible communication tools; involve families early in transition planning; offer genetic counseling
Bardet-Biedl syndrome	Obesity, intellectual disability, retinal dystrophy, genitourinary anomalies	Cognitive impairment; limited self-management capacity	Provide structured transition training; tailor health literacy materials; offer reproductive and genetic counseling

### Systemic and structural barriers to transition in rare diabetes

The transition from pediatric to adult care presents considerable systemic challenges for AYA with rare forms of diabetes (Box 1). These challenges are rooted both in general structural differences between healthcare systems and in specific barriers related to the complexity of rare diseases.

Box 1 **Summary of transition barriers in rare diabetes**

- Diagnostic delay and misclassification (e.g., MODY misdiagnosed as type 1 diabetes or type 2 diabetes).
- Lack of structured, multidisciplinary transition models.
- Limited adult provider expertise in rare diabetes syndromes.
- Progressive sensory or neurocognitive impairments (e.g., Wolfram, BBS).
- Poor self-efficacy and low health literacy among patients.
- Inadequate caregiver preparation for shared decision-making.
- Fragmented care coordination between pediatric and adult systems.

#### Lack of standardized transition protocols

A major systemic gap lies in the absence of standardized, evidence-based transition pathways for rare diabetes syndromes. Unlike more prevalent chronic conditions, such as type 1 diabetes, where structured models (e.g., transition clinics and readiness assessments) have been more broadly implemented, rare syndromic diabetes often lacks formalized guidance. This leads to variability in timing, preparation, and responsibility for initiating the transition process (8, 12, 22, 23).

Moreover, many adult endocrinology services are unfamiliar with the specific needs of this patient population, particularly in regions without specialized rare disease centers. As a result, transition is often ad hoc and fragmented, with patients ‘aging out’ of pediatric care rather than being actively transferred with a tailored handover plan.

#### Poor interdisciplinary coordination

Effective transition in rare diabetes requires ongoing collaboration between multiple disciplines, including pediatric and adult endocrinologists, geneticists, nephrologists, ophthalmologists, audiologists, psychologists, and social workers. However, interdisciplinary interfaces between pediatric and adult services are frequently underdeveloped or absent. Poor communication and lack of shared medical summaries or case conferences can result in redundant testing, missed complications, or inconsistent therapy (24, 25, 26).

This is particularly problematic for syndromic cases (e.g., WS, AS, BBS), where care must extend beyond diabetes management to include multisystem surveillance and supportive services. In most countries, these services are dispersed across multiple institutions or providers, increasing the burden on families to coordinate care themselves.

#### Limited availability of multidisciplinary transition clinics

Dedicated transition clinics – where pediatric and adult teams meet jointly with patients – have been shown to improve readiness, treatment adherence, and follow-up continuity in other chronic conditions. However, such structures are still rare in the field of rare endocrine diseases. Only a few expert centers across Europe offer integrated transition pathways for rare diabetes syndromes (8, 27).

The lack of such models disproportionately affects patients with neurocognitive impairments or sensory disabilities, for whom consistent relationships and clear communication structures are crucial for engagement and safety.

#### Structural differences between pediatric and adult systems

Pediatric care is typically centralized, multidisciplinary, and family-centered, with proactive case management by pediatric endocrinologists and support from nurses, educators, psychologists, and therapists. This model ensures coordinated, long-term follow-up, often within tertiary care centers.

In contrast, adult healthcare is more fragmented and reactive, with limited coordination and a focus on organ-specific issues. AYA are expected to independently manage appointments and advocate for their care – expectations that may exceed their capacities, especially in the context of rare, complex conditions.

This shift can lead to the loss of trusted providers, discontinuation of tailored monitoring or assistive technologies, and gaps in therapy access due to insurance or formulary differences.

#### Caregiver readiness and shared decision-making

An often-overlooked barrier in rare diabetes transition is the insufficient preparation of caregivers for their evolving role during adolescence. In many cases, caregivers – especially parents – remain deeply involved in coordinating care and managing complex regimens throughout childhood. However, they are rarely included systematically in the transition planning process or supported in handing over responsibilities to their adolescent children.

This lack of preparation may hinder effective shared decision-making, as families struggle to balance autonomy building with ongoing support. Particularly in syndromic conditions involving neurocognitive or sensory impairments, a gradual and supported shift in responsibility is essential to avoid overburdening either party. Structured family counseling, communication training, and role clarification are needed to facilitate this process (14, 23, 28).

#### Consequences of systemic disruption

These system-level mismatches increase the risk of:

- Loss to follow-up, especially for patients with limited self-management capacity.
- Worsening metabolic control due to therapy discontinuation or mismanagement.
- Missed comorbidities, such as hearing loss, retinopathy, or nephropathy, which may not be systematically screened in adult clinics.
- Psychosocial distress, as adolescents feel unprepared or unsupported in the adult care environment.

To address these gaps, health systems must prioritize the development of cross-sectoral, longitudinal care pathways, ideally anchored in expert centers with outreach or telehealth capacity for shared care models. Where joint clinics are not feasible, structured referral, shared care plans, and transition coordinators can mitigate the risk of discontinuity.

### Psychosocial and adherence challenges

Psychological readiness, self-efficacy, and health literacy are often lower in AYA with rare syndromes, particularly those with neurocognitive impairments or visual/hearing deficits. This increases the risk of nonadherence, loss to follow-up, and worsening metabolic control during transition (23, 29, 30, 31). AYA with WS and BBS are especially vulnerable due to progressive sensory and cognitive decline.

In addition, parents and caregivers frequently play a central role in managing care throughout childhood, and insufficient caregiver engagement during transition planning can further compromise outcomes (14). Social stigma, lack of peer support, and the emotional burden of managing a lifelong rare disease further exacerbate psychosocial stressors during this period.

## Collaborative transition models and network initiative

Despite increasing recognition of the need for structured transition programs, the implementation and quality of transition practices for adolescents with rare forms of diabetes remain inconsistent across Europe and globally. Various international initiatives – including Endo-ERN, SWEET, and ISPAD – have developed recommendations, but translation into practice varies widely depending on local resources, training, and institutional infrastructure.

### International initiatives supporting transition

The European Reference Network on Rare Endocrine Conditions (Endo-ERN) has placed increasing focus on structured transition, including its Transition of Care (ToC) Working Group and several initiatives from or in collaboration with Main Thematic Group 3 (MTG3), which is dedicated to rare genetic disorders affecting glucose and insulin homeostasis (MTG3 Overview – Endo-ERN). These efforts aim to harmonize care pathways for patients with rare endocrine and metabolic conditions. However, a recent review of Endo-ERN centers identified significant heterogeneity in the availability of transition clinics, formal protocols, and documentation of transition readiness (32). Several networks have taken steps to address these gaps. Endo-ERN, particularly MTG3, is focused on genetic syndromes of glucose and insulin homeostasis. The International Society for Pediatric and Adolescent Diabetes (ISPAD) has released consensus statements on transition and completed a global survey of current practices (3, 33). The SWEET registry supports quality benchmarking in pediatric diabetes care and has started to integrate transition-related metrics (34).

The SWEET registry and network, which gather longitudinal data on pediatric diabetes care from certified centers of excellence facilitating benchmarking, have integrated transition metrics and carried out a survey among all centers about transition and a project based on data analysis is underway to identify the state of the art of transition, among SWEET centers worldwide. Still, most data pertain to type 1 diabetes, with limited representation of monogenic or syndromic forms of diabetes (35).

ISPAD has published transition guidelines and conducted surveys among members to assess current practices. Results show a wide range of practices and often a lack of disease-specific pathways for rare diabetes syndromes (36). ISPAD promotes patient-centered transition, but adoption of specific rare disease approaches remains an area for growth.

## Proposed strategies and frameworks for improving transition in rare diabetes

Improving transition for AYA with rare diabetes syndromes requires an integrated, longitudinal model of care tailored to complex multisystem needs. Structured transition should be distinguished from the mere act of transfer; it must begin early, include readiness assessment, and extend well into adult care.

### Models of coordinated, multidisciplinary care

Successful transition hinges on collaborative planning between pediatric and adult providers, incorporating syndrome-specific expertise across endocrinology, nephrology, audiology, ophthalmology, neurology, and psychology. While joint visits between pediatric and adult teams can facilitate handover, such models are not feasible in all settings. Instead, structured, digitally supported communication and shared documentation (e.g., standardized transition summaries) should be core components (12, 21).

### Patient-centered, resource-oriented transition frameworks

**Figure 1:**
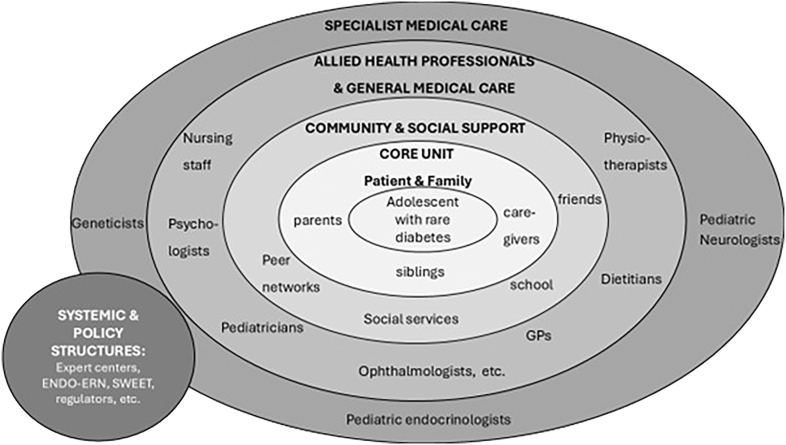
Resource-oriented model of transition care for AYA with rare diabetes. This concentric model illustrates the layered support structures surrounding an AYA with a rare diabetes syndrome. At the core are the patient and their family, including parents and close social contacts. The second layer represents community and social support networks, such as schools, caregivers, and peer connections. Allied health professionals and general medical care providers, including pediatricians, dietitians, psychologists, and general practitioners, form the third layer. Specialist medical care – such as pediatric diabetologists, endocrinologists, neurologists, and geneticists – is positioned next, providing disease-specific expertise. The outermost layer includes systemic and policy-level structures, such as expert centers, the Endo-ERN and SWEET networks, and regulatory authorities. The model emphasizes the need for coordinated, multilayered engagement to ensure successful transition from pediatric to adult care (37).

Pediatric care typically follows a resource-oriented model where allied professionals, families, and school networks are actively engaged in care coordination. Adult care, by contrast, often lacks these systemic supports. Figure 1 illustrates a concentric, resource-oriented model of transition, emphasizing the need for a multilayered system involving families, specialists, community services, and policy structures (37).

### Structured transition pathways

**Figure 2:**
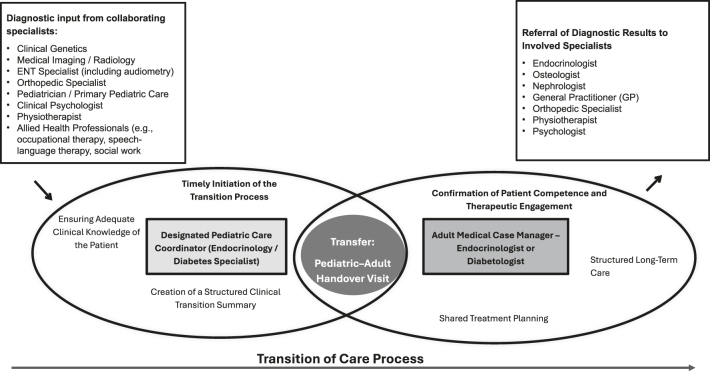
Structured transition of care process for AYA with rare forms of diabetes. This diagram outlines the key stages and responsible stakeholders involved in the transition from pediatric to adult care for AYA with rare diabetes syndromes. The process includes timely initiation, designation of pediatric and adult care coordinators, creation of a clinical summary, assessment of patient competence, and structured referral to involved specialists. The aim is to ensure continuity, multidisciplinary collaboration, and patient-centered care throughout the transition phase.

Effective transition requires defined roles and communication strategies. Pediatric care teams must initiate the process by mid-adolescence, including goal setting, syndrome-specific recommendations, and competence assessment. Adult providers should be prepared to receive detailed summaries and integrate relevant specialists. Transition coordinators or case managers play a vital role in ensuring continuity. These steps are captured in Fig. 2.

### PROMs, digital tools, and quality of life (QoL) assessment in rare diabetes transition

To enable effective and patient-centered transition, structured tools for assessing transition readiness, psychosocial well-being, and QoL are critical, particularly for AYA with syndromic or multisystem diabetes.

### Transition readiness and PROMs

Validated instruments help identify gaps in knowledge, autonomy, and support needs during transition planning. The Transition Readiness Assessment Questionnaire (TRAQ) is widely used across chronic conditions to evaluate preparedness and self-management skills (38). Disease-agnostic tools such as TRANSITION-Q and Mind the Gap have been successfully adapted for youth with complex health conditions, allowing for personalized interventions (28, 39).

In addition, disease-specific PROMs are needed to capture cognitive, emotional, and sensory aspects in syndromes such as Wolfram or BBS. These tools support shared decision-making, inform care planning, and can serve as outcome metrics across networks such as SWEET or Endo-ERN. In this context, the READDY (Rare Endocrine and Diabetes Disorders in Youth) project aims to collect real-world data on transition practices, access to care, and patient-reported outcomes across European centers. Inspired by the increasing use of structured transition readiness tools such as the READDY questionnaire developed by Corathers *et al.* (40) in the USA, the European READDY initiative – carried out within the Endo-ERN framework – aims to systematically assess transition processes and barriers in rare endocrine and diabetes care across Europe. While not directly related, both share the goal of improving structured, needs-based transition pathways and informing future policy development.

### Quality of life (QoL) assessment

QoL evaluations are essential in rare diabetes syndromes marked by progressive disability. Instruments such as DISABKIDS (41) and PedsQL 4.0 (42) allow comparison across populations and can be applied across pediatric and young adult age groups. Screening for mental health conditions should also be integrated into routine transition assessment. Recommended tools include WHO-5, PHQ-4/8, and GAD-7, which are validated for adolescents and widely available in multiple languages (43, 44, 45).

### Digital tools, education platforms, and accessibility

Emerging digital technologies offer promising avenues for supporting structured transition. These include:

Transition apps and online portals (e.g., digital passports and symptom trackers) that support patient empowerment and information continuity (46), and telemedicine and virtual joint visits, especially relevant for patients with sensory disabilities or limited access to expert centers (47).

Online education platforms designed for AYA and caregivers to promote health literacy and condition-specific knowledge.

These tools help bridge the structural gap between pediatric and adult care, support communication, and expand access to specialized transition resources. Reference centers and ERNs should ensure these services are accessible to all patients, irrespective of geographic location or functional impairment.

Furthermore, international registries such as SWEET and networks such as Endo-ERN are encouraged to systematically integrate PROMs, QoL metrics, and patient satisfaction outcomes into their benchmarking and quality frameworks to ensure transition quality is not only documented but actively improved.

## Health system considerations and financing of transition programs

Sustainable transition processes for adolescents with rare diabetes syndromes require not only clinical coordination and educational efforts but also adequate financial and structural support. Despite increasing awareness, many health systems lack dedicated reimbursement pathways or funding models to support structured transition services, especially those that involve multidisciplinary care, case management, and cross-sectoral coordination.

### Financial gaps and system barriers

Current reimbursement systems in many countries are primarily designed around acute care or adult-oriented chronic care management, with limited flexibility to cover preparatory transition activities in pediatric settings or follow-up coordination in adult care (48, 49). This is particularly problematic for rare diseases, where individualized care plans and additional time for education, psychosocial support, and digital tools are often needed.

### Need for dedicated funding structures

Evidence from transition programs in chronic diseases suggests that structured interventions – such as dedicated transition coordinators, interdisciplinary meetings, or joint clinics – improve care continuity and outcomes but require appropriate funding streams (50). Policymakers should consider the introduction of bundled payment models, time-based billing codes, or transition-specific care pathways within insurance frameworks to support these services.

### European and international support structures

At the European level, reference networks such as Endo-ERN could play a pivotal role in advocating for equitable access to funded transition programs across member states. Transition funding should also be included in national rare disease plans and supported through strategic public health initiatives, such as those promoted by the European Joint Action on Rare Diseases (JARDIN) and national rare disease alliances.

### Call to action

To reduce inequalities and avoid long-term healthcare costs associated with poor transition outcomes – such as loss to follow-up, emergency care use, or deterioration of chronic conditions – investments in early, structured, and sustained transition processes are urgently needed.

## Patient perspectives and lessons from the field

Real-life scenarios in transitioning AYA with rare diabetes syndromes provide crucial insights into both barriers and best practices. The heterogeneity of national care systems and the complexity of syndromic conditions such as AS, BBS, and WS amplify the need for individualized, well-coordinated transition strategies.

### Case 1: Successful transition in a patient with BBS

A 16-year-old with BBS, managed at a tertiary pediatric center, had received multidisciplinary care from early childhood, including endocrinology, nephrology, ophthalmology, and psychological support. At age 14, a transition coordinator was introduced to initiate longitudinal planning. A transition readiness assessment was completed annually, and the adolescent and their family were gradually introduced to the adult team via joint visits.

Adapted educational materials were provided to accommodate cognitive limitations, and the patient was actively involved in developing a personal health passport. After transfer at age 17, regular follow-ups were maintained with the adult team, and support from a diabetes nurse educator continued uninterrupted.

**Caregiver voice:** ‘We felt heard every step of the way. Knowing there was a plan, and that the adult providers understood not just the diabetes, but the syndrome, gave us great peace of mind’.

**Key success factors:** early preparation, caregiver inclusion, syndrome-specific training for adult teams, and stable care continuity.

### Case 2: Fragmented transition in a patient with WS

A 17-year-old with WS was transferred to adult care with minimal preparation. Despite receiving complex pediatric care involving diabetology, ophthalmology, endocrinology, audiology, and neurology, no structured transition planning occurred. The adolescent had progressive vision and hearing loss and mild cognitive delay, yet no accommodations were made for communication needs.

At the transfer visit, the adult endocrinologist had not received the full medical summary, and no joint consultation occurred. Within 6 months, the patient missed multiple appointments due to logistical confusion and disengagement.

**Parent voice:** ‘It felt like the system just dropped us. The new doctors didn’t know what Wolfram was. We had to explain everything ourselves’.

**Key challenges:** lack of preparatory planning, absence of inter-team communication, no joint visits, and insufficient caregiver support.

### Lessons learned

- Transition planning must begin early and be adapted to cognitive and sensory needs.
- Joint consultations build trust, ensure information continuity, and support shared decision-making.
- Digital tools and transition coordinators enhance cross-disciplinary care alignment.
- Successful transitions require both clinical and emotional preparedness for patients and families.
- Listening to caregiver perspectives helps identify system gaps and refine transition pathways.

## Conclusions and policy implications

The transition from pediatric to adult care represents a critical juncture for adolescents with rare forms of diabetes. Syndromes such as MODY, WS, BBS, and AS are characterized by multisystem involvement, progressive complications, and often cognitive or dual sensory impairments. These factors significantly increase the risks associated with inadequate or poorly structured transitions.

Our review emphasizes that current transition practices are often fragmented, generic, or absent altogether, failing to reflect the complexity of rare diabetes syndromes. Despite promising efforts by international networks such as Endo-ERN, ISPAD, SWEET, and conect4children (c4c), there remains substantial heterogeneity in clinical practice, insufficient cross-specialty coordination, and limited use of structured assessment tools such as PROMs or digital care platforms.

We advocate for the development and implementation of syndrome-aware, consensus-based international transition frameworks for rare diabetes forms. While current evidence remains limited due to the ultra-rare nature of these conditions, expert knowledge, registry data, and patient perspectives can guide the creation of adaptable, multidisciplinary care pathways. These frameworks should be co-designed with young people, families, and patient advocacy groups, and embedded in the strategic agendas of Endo-ERN, ESE, ESPE, and ISPAD. A focus on continuity, psychosocial needs, digital inclusion, and care coordination is essential to promote equity and long-term outcomes. Policy interventions at the national and EU level are urgently needed. This includes:

- Ensuring universal access to multidisciplinary transition services;
- Supporting the training of adult healthcare providers in rare endocrine disorders;
- Facilitating longitudinal care pathways that extend beyond a single transfer encounter;
- Revising medical device regulations and reimbursement frameworks to accommodate the specific needs of pediatric and transition-age populations, particularly for technologies such as insulin pumps and continuous glucose monitoring (CGM) systems.
- Sustainable transition care requires dedicated funding as part of standard rare disease management.

This is a pivotal moment to shift the paradigm from transition as a handover to transition as a shared, structured, and individualized process. By doing so, we can safeguard the health, autonomy, and long-term outcomes of adolescents living with rare forms of diabetes.

## Declaration of interest

The authors declare that there is no conflict of interest that could be perceived as prejudicing the impartiality of the work reported.

## Funding

This work was developed within the framework of the Transition of Care Working Group of the European Reference Network on Rare Endocrine Conditions (Endo-ERN). No specific external funding was received for the preparation of this manuscript.
